# Effect of the dietary supplement Meltdown on catecholamine secretion, markers of lipolysis, and metabolic rate in men and women: a randomized, placebo controlled, cross-over study

**DOI:** 10.1186/1476-511X-8-32

**Published:** 2009-08-05

**Authors:** Richard J Bloomer, Robert E Canale, Megan M Blankenship, Kelley G Hammond, Kelsey H Fisher-Wellman, Brian K Schilling

**Affiliations:** 1 Cardiorespiratory/Metabolic Laboratory, Department of Health and Sport Sciences, University of Memphis, Memphis, TN, USA

## Abstract

**Background:**

We have recently reported that the dietary supplement Meltdown^® ^increases plasma norepinephrine (NE), epinephrine (EPI), glycerol, free fatty acids (FFA), and metabolic rate in men. However, in that investigation measurements ceased at 90 minutes post ingestion, with values for blood borne variables peaking at this time. It was the purpose of the present investigation to extend the time course of measurement to 6 hours, and to include women within the design to determine if sex differences to treatment exist.

**Methods:**

Ten men (24 ± 4 yrs) and 10 women (22 ± 2 yrs) ingested Meltdown^® ^or a placebo, using a randomized, cross-over design with one week separating conditions. Blood samples were collected immediately before supplementation and at one hour intervals through 6 hours post ingestion. A standard meal was provided after the hour 3 collection. Samples were assayed for EPI, NE, glycerol, and FFA. Five minute breath samples were collected at each time for measurement of metabolic rate and substrate utilization. Area under the curve (AUC) was calculated. Heart rate and blood pressure were recorded at all times. Data were also analyzed using a 2 (sex) × 2 (condition) × 7 (time) repeated measures analysis of variance, with Tukey *post hoc *testing.

**Results:**

No sex × condition interactions were noted for AUC for any variable (p > 0.05). Hence, AUC data are collapsed across men and women. AUC was greater for Meltdown^® ^compared to placebo for EPI (367 ± 58 pg·mL^-1^·6 hr^-1 ^vs. 183 ± 27 pg·mL^-1^·6 hr^-1^; p = 0.01), NE (2345 ± 205 pg·mL^-1^·6 hr^-1 ^vs. 1659 ± 184 pg·mL^-1^·6 hr^-1^; p = 0.02), glycerol (79 ± 8 μg·mL^-1^·6 hr^-1 ^vs. 59 ± 6 μg·mL^-1^·6 hr^-1^; p = 0.03), FFA (2.46 ± 0.64 mmol·L^-1^·6 hr^-1 ^vs. 1.57 ± 0.42 mmol·L^-1^·6 hr^-1^; p = 0.05), and kilocalorie expenditure (439 ± 26 kcal·6 hrs^-1 ^vs. 380 ± 14 kcal·6 hrs^-1^; p = 0.02). No effect was noted for substrate utilization (p = 0.39). Both systolic and diastolic blood pressure (p < 0.0001; 1–16 mmHg), as well as heart rate (p = 0.01; 1–9 bpm) were higher for Meltdown^®^. No sex × condition × time interactions were noted for any variable (p > 0.05).

**Conclusion:**

Ingestion of Meltdown^® ^results in an increase in catecholamine secretion, lipolysis, and metabolic rate in young men and women, with a similar response for both sexes. Meltdown^® ^may prove to be an effective intervention strategy for fat loss, assuming individuals are normotensive and their treatment is monitored by a qualified health care professional.

## Background

The prevalence of obesity has increased to epidemic proportions in recent years, with 400 million individuals classified as obese worldwide [1,2] defined as having a body mass index ≥ 30 kg·m^-2^. An additional 1.6 billion individuals are currently classified as overweight worldwide [2] defined as having a body mass index between 25 and 29.9 kg·m^-2^. Over the counter (OTC) dietary supplements are often used as an aid in body fat/weight loss. Unfortunately, many such supplements have little to no scientific support in human subjects, while some have been reported to cause ill-health [3]. Specifically, many products rely exclusively on research which is conducted using the "key ingredient" within the product of sale, rather than the actual finished product, often at dosages that are much higher than what is used in the actual product of sale. Despite this shortcoming, the dietary supplement market reached nearly $20 billion in United States sales in 2007, according to the Nutrition Business Journal, and for weight loss agents alone was estimated to be a 700 million dollar industry in 2008 [4].

Although many isolated ingredients have been shown to have promise in relation to lipolysis, three that have been well studied and included in many OTC dietary supplements currently sold on the market include yohimbine [5], synephrine [6], and caffeine [7]. The specific mechanisms of action of these ingredients have been presented in our recently published paper [8]. Novel variants of these ingredients have been combined into a single dietary supplement (Meltdown^®^).

We have recently reported that the finished product Meltdown^® ^results in a significant increase in the area under the curve (AUC) for blood norepinephrine (NE), glycerol, and free fatty acids (FFA), in addition to a significant increase in metabolic rate compared to placebo [8]. However, treatment with this agent produced an increase in heart rate (5–6 bpm) and blood pressure (6–10 mmHg) within the majority of subjects, which was of statistical significance for systolic blood pressure (p = 0.04). This latter finding may be of concern for those with elevated blood pressure. This is an important consideration in the regular use of any dietary weight loss product, as many contain stimulants which may promote a significant hemodynamic response [9]. However, in this initial work, measurements were only made for 90 minutes post ingestion (pre, 30 min, 60 min, 90 min), with blood values for all variables peaking at the 90 minute post ingestion time. These data indicate that a longer time course of measurement is needed to more fully evaluate the potential lipolytic effects of this dietary supplement, as well as to better assess the hemodynamic effects of treatment. Assuming a more prolonged effect, this agent may prove efficacious in the treatment of obesity.

Aside from our failure to include a longer time course of measurement, our initial work used men exclusively as subjects. Therefore, it is presently unknown whether or not women respond to this treatment in the same manner as do men. It is possible that men and women may respond differently to supplementation [10], thus generalizing our initial findings in which we used men exclusively as subjects [8] may be problematic. Moreover, because many women are regular consumers of OTC dietary weight loss supplements, it is important to understand how they would respond to such treatment. Therefore, the purpose of the present investigation was to extend our prior findings and to study the impact of the lipolytic agent Meltdown^® ^on blood catecholamines, markers of lipolysis, and metabolic rate in men and women over the course of a six hour post ingestion period, while also monitoring the hemodynamic response to treatment.

## Methods

### Subjects

Healthy, exercise-trained men (n = 10) and women (n = 10) participated in this investigation. Subjects completed a medical history and physical activity questionnaire to determine eligibility. No subject was a smoker or had diagnosed metabolic (including pre-diabetes or diabetes) or cardiovascular disease. Both men and women were considered to be exercise-trained, as they performed combined aerobic and anaerobic exercise for 8 ± 3 and 8 ± 4 hrs per week, respectively, for the past several years. Subject descriptive characteristics are presented in Table 1. All experimental procedures were performed in accordance with the Helsinki Declaration. The University of Memphis Human Subjects Committee approved all experimental procedures. All subjects provided both verbal and written consent prior to participating in this study.

**Table 1:** Descriptive characteristics of subjects

Variable	Men (n = 10)	Women (n = 10)	P value
Age (yrs)	23.7 ± 4.3	21.9 ± 2.4	0.2636
Height (cm)	177.2 ± 4.7	167.4 ± 7.2	0.0019
Weight (kg)	77.4 ± 6.7	63.6 ± 11.1	0.0033
BMI (kg·m^-2^)	24.8 ± 2.9	22.6 ± 2.8	0.1063
Body fat (%)*	8.8 ± 3.3	23.0 ± 5.9	0.0001
Waist (cm)	81.5 ± 5.9	70.4 ± 6.2	0.0007
Hip (cm)	99.1 ± 3.0	99.4 ± 7.4	0.9225
Waist:Hip	0.82 ± 0.06	0.71 ± 0.03	0.0001
Years Anaerobic Exercise	6.2 ± 5.4	3.3 ± 3.3	0.1584
Hours per week Anaerobic Exercise	4.7 ± 2.5	3.1 ± 3.3	0.2396
Years Aerobic Exercise	3.9 ± 3.3	4.7 ± 4.0	0.6525
Hours per week Aerobic Exercise	3.1 ± 2.5	4.7 ± 3.5	0.2698

Data are mean ± SD.

* Determined from 7-site skinfold analysis use Lange calipers and Siri equation

### Conditions and Testing

**Figure 1:**
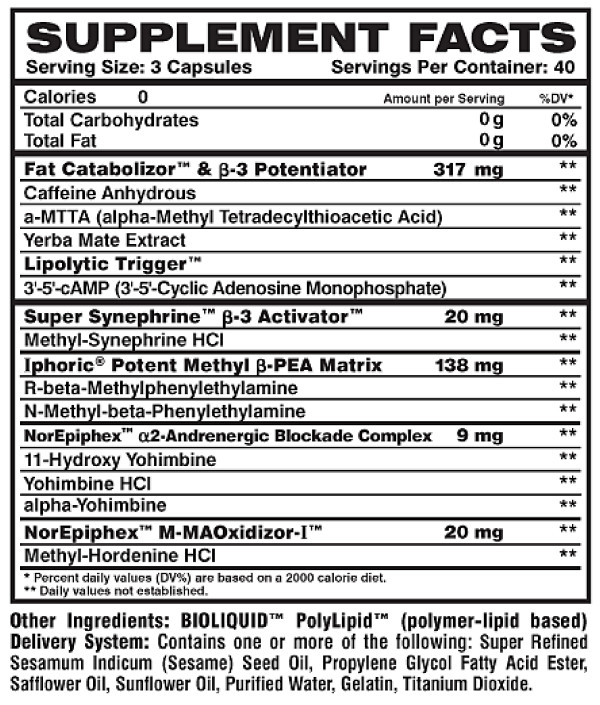
**Label description of Meltdown^®^**.

Procedures described below were identical for both test sessions (supplement and placebo). The dietary supplement used in this investigation (Meltdown^®^; Vital Pharmaceuticals, Inc., Davie, FL) included yohimbine, caffeine, and synephrine as the primary active ingredients. Please see Figure 1 for a description of the dietary supplement. Capsules were from the same bottle and produced in accordance with Good Manufacturing Practices (GMPs). Prior to production, all raw materials were tested for ingredient potency and the finished product was verified for label claims. Subjects consumed the upper limit of the recommended dosage of the dietary supplement (as stated on the product label), which consisted of three capsules, or an identical looking placebo (corn starch, microcrystalline cellulose, super refined sesame oil, propylene glycol fatty acid ester, safflower oil, sunflower oil). It should be noted that the label states that this product is "Not for use by individuals under the age of 18, by those with a medical condition, or by pregnant or nursing women". The experiment was conducted in a double blind, cross-over design. Please note that due to the potency of the dietary supplement and the fact that it contains stimulants, many subjects reported that they could "feel" an effect. Hence, the double blind nature of the design might be considered questionable. A standard meal replacement bar (Zero Impact^®^; Vital Pharmaceuticals, Inc., Davie, FL) was provided to subjects after the three hour collection period. The 112 gram bar consisted of 440 kcal, 30 grams of protein, 35 grams of carbohydrate, and 20 grams of fat. Subjects were instructed to consume as much of the bar as desired. Any amount not consumed was weighed on a laboratory grade balance, and this determined the amount of bar consumed. The same amount was provided to subjects during their second test session. Female subjects consumed a mean of 62 grams of the 112 gram bar, while male subjects consumed a mean of 102 grams of the 112 gram bar. No other food was allowed during the six hour period. However, water was allowed ad libitum, and was measured and matched for both days of testing (mean intake for women = 1921 mL; mean intake for men = 2163 mL).

Subjects reported to the laboratory in a fasted state (>8 hours), without caffeine consumption during the previous 8 hours. All testing was started by 1100 hours and the time for each subject was matched for both visits. Subjects were asked not to exercise for the 48 hours prior to each testing day. Women reported during the first nine days of their menstrual cycle in order to avoid any potential influence of estrogen on our chosen outcome measures. Following a 10 minute quiet rest period, heart rate (via monitor) and blood pressure (via auscultation) were measured, a blood sample was obtained, and subjects provided a five minute breath sample (for analysis of metabolic rate). Subjects then ingested either the supplement or placebo, in the presence of an investigator. At the start of each one hour period, the same exact collection procedure as described above was followed. Therefore, these variables were collected a total of seven times for all subjects (pre-ingestion, 1, 2, 3, 4, 5, 6 hours post-ingestion). Subjects remained inactive in the laboratory during the entire six hour test period.

The measurement of metabolic rate was performed using indirect calorimetry via breath-by-breath collection (SensorMedics Vmax 229 metabolic system; Yorba Linda, CA). All gas collection took place in a temperature and humidity controlled laboratory, and both the flow sensor and gas analyzers were calibrated prior to data collection. Total oxygen consumption (L·min^-1^) was determined and total kilocalorie expenditure was estimated from this value. Respiratory exchange ratio (RER) was also determined from gas collection (CO_2_/O_2_), and used as a crude measure of substrate utilization.

### Blood Collection and Biochemistry

A total of seven venous blood samples (7 mL per draw) were taken from subjects' forearm via needle and Vacutainer^®^. Blood was immediately processed in a refrigerated centrifuge in order to obtain plasma (4°C for 15 min at 2000 × g). Plasma samples were stored in multiple aliquots at -80°C. All assays were performed on first thaw within six weeks of sample collection. NE and EPI were determined using an enzyme linked immunosorbent assay (2-CAT ELISA, BA 10–1500; Rocky Mountain Diagnostics) following the instructions of the manufacturer (Labor Diagnostika Nord GmbH & Co. KG). In this competitive ELISA, NE and EPI are extracted by using a cis-diol-specific affinity gel, acylated, and then derivitized enzymatically. The coefficient of variation (CV) for NE and EPI was 8.8% and 7.5%, respectively. Glycerol was determined using the Free Glycerol Determination Kit (FG0100) and Glycerol Standard (G7793), following the instructions of the manufacturer (Sigma Aldrich). The CV for glycerol was 7.4%. Free fatty acids were determined using the Free Fatty Acid Quantification Kit (K612-100) following the instructions of the manufacturer (BioVision). The CV for FFA was 8.5%.

### Diet and Physical Activity

During the 24 hours before each test day, subjects consumed prepackaged meal replacement drinks (protein-rich ready-to-drink shake; Vital Pharmaceuticals, Inc., Davie, FL) and food bars (Zero Impact^®^; Vital Pharmaceuticals, Inc., Davie, FL). These contained a mix of protein, carbohydrate, and fat. Subjects were each provided with 3 shakes and 3 bars and instructed to consume as many as they desired. No other food or calorie containing drinks were allowed. The amount consumed during the day preceding the initial test day was mimicked during the day preceding the second test day. The mean intake for men was 3 shakes and 2.5 bars, while for women this was 2 shakes and 1.5 bars. These amounts provided approximately 2000 kilocalories to men and 1300 kilocalories to women. While these amounts were estimated to be 500–750 kilocalories lower than subjects' usual intake (based on subjects' reports), satiety with use of these meal replacements was reported by most subjects to be acceptable.

### Statistical Analysis

Area under the curve (AUC) was calculated for each biochemical and metabolic variable for both conditions using the trapezoidal method (AUC_G_) as described in detail by Pruessner et al. [11]. Statistical comparisons for biochemical (AUC_G_) and metabolic data were made using a 2 (sex) × 2 (condition) repeated measures analysis of variance (RMANOVA). In addition, biochemical, metabolic, and hemodynamic data were also compared using a 2 (sex) × 2 (condition) × 7 (time) RMANOVA. Tukey's *post hoc *testing was used where appropriate. All analyses were performed using JMP statistical software (version 4.0.3, SAS Institute, Cary, NC). Statistical significance was set at P = 0.05. The data are presented as mean ± SEM, except for subject descriptive characteristics (mean ± SD).

## Results

### Biochemical Data

**Figure 2:**
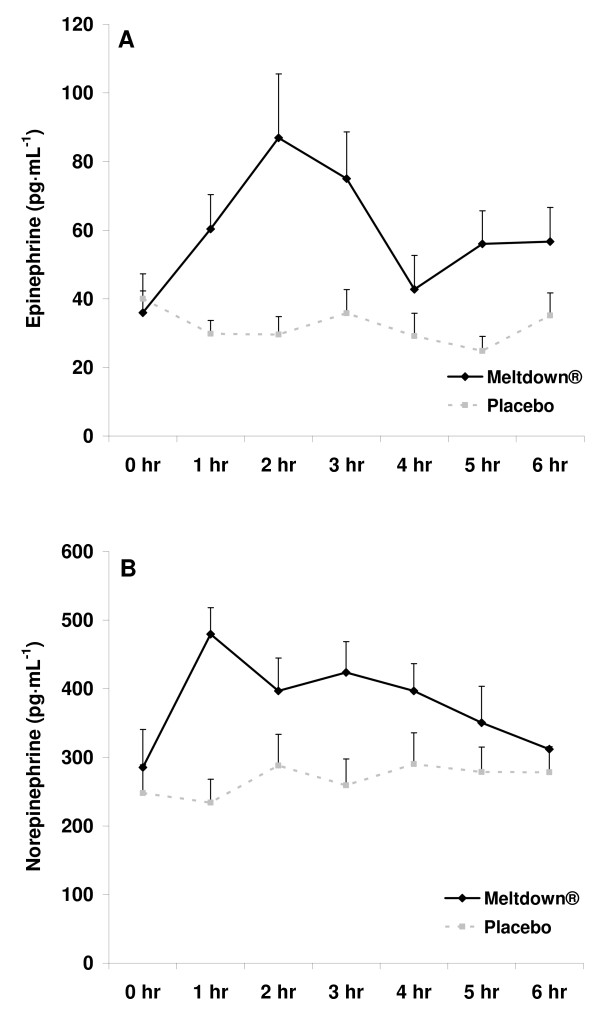
**Plasma epinephrine (A) and norepinephrine (B) data for men and women consuming Meltdown^® ^and placebo in a randomized cross-over design**. Data are mean ± SEM. * Greater epinephrine (367 ± 58 pg·mL^-1^·6 hr^-1 ^vs. 183 ± 27 pg·mL^-1^·6 hr^-1^; p = 0.01) and norepinephrine (2345 ± 205 pg·mL^-1^·6 hr^-1 ^vs. 1659 ± 184 pg·mL^-1^·6 hr^-1^; p = 0.02) AUC for Meltdown^® ^compared to placebo.

**Figure 3:**
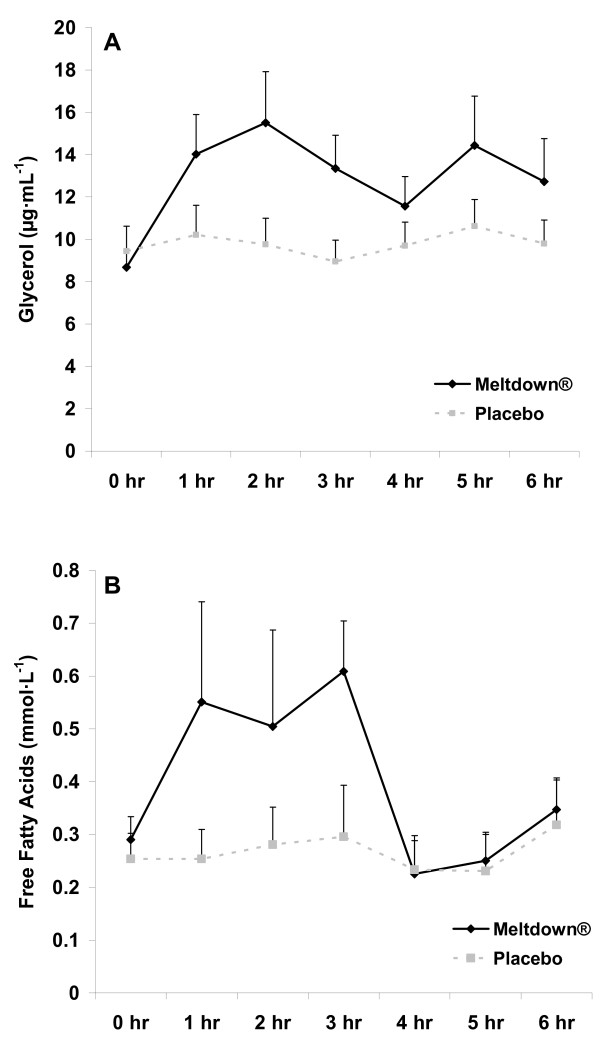
**Plasma glycerol (A) and free fatty acid (B) data for men and women consuming Meltdown^® ^and placebo in a randomized cross-over design**. Data are mean ± SEM. * Greater glycerol (79 ± 8 μg·mL^-1^·6 hr^-1 ^vs. 59 ± 6 μg·mL^-1^·6 hr^-1^; p = 0.03), and FFA (2.46 ± 0.64 mmol·L^-1^·6 hr^-1 ^vs. 1.57 ± 0.42 mmol·L^-1^·6 hr^-1^; p = 0.05) AUC for Meltdown^® ^compared to placebo.

All subjects successfully completed all aspects of the study. Expected differences were noted between men and women for selected anthropometric variables (Table 1). When considering the AUC analysis, no sex × condition interactions were noted for EPI (p = 0.57), NE (p = 0.58), glycerol (p = 0.39), or FFA (p = 0.37). Likewise, when considering the 2 (sex) × 2 (condition) × 7 (time) RMANOVA, no three way interactions were noted for any variable (p > 0.05). Therefore, data are presented as pooled data for men and women in Figures 2 and 3.

Regarding the AUC main effect analyses, a condition effect was noted for EPI (p = 0.01; Figure 2A), with no sex effect noted (p = 0.11). The mean EPI AUC response for Meltdown^® ^compared to placebo was 96% for men and 91% for women. Values increased across time with Meltdown^® ^and were higher at 1, 2, and 3 hours post ingestion compared to pre-ingestion (p < 0.05). Both a condition (p = 0.02; Figure 2B) and sex (p = 0.01) effect was noted for NE, with values higher for men compared to women. The mean NE AUC response for Meltdown^® ^compared to placebo was 44% for men and 42% for women. Values increased across time with Meltdown^® ^and were higher at 1 and 3 hours post ingestion compared to pre-ingestion (p < 0.05). Both a condition (p = 0.03; Figure 3A) and sex (p = 0.01) effect was noted for glycerol, with values higher for women compared to men. The mean glycerol AUC response for Meltdown^® ^compared to placebo was 25% for men and 42% for women. Values increased across time with Meltdown^® ^and were higher at 1, 2, and 3 hours post ingestion compared to pre-ingestion (p < 0.05). Both a condition (p = 0.05; Figure 3B) and sex (p = 0.05) effect was noted for FFA, with values higher for women compared to men. The mean FFA AUC response for Meltdown^® ^compared to placebo was 21% for men and 35% for women. Values increased across time with Meltdown^® ^and were higher at 1 and 3 hours post ingestion compared to pre-ingestion (p < 0.05).

### Metabolic Data

**Figure 4:**
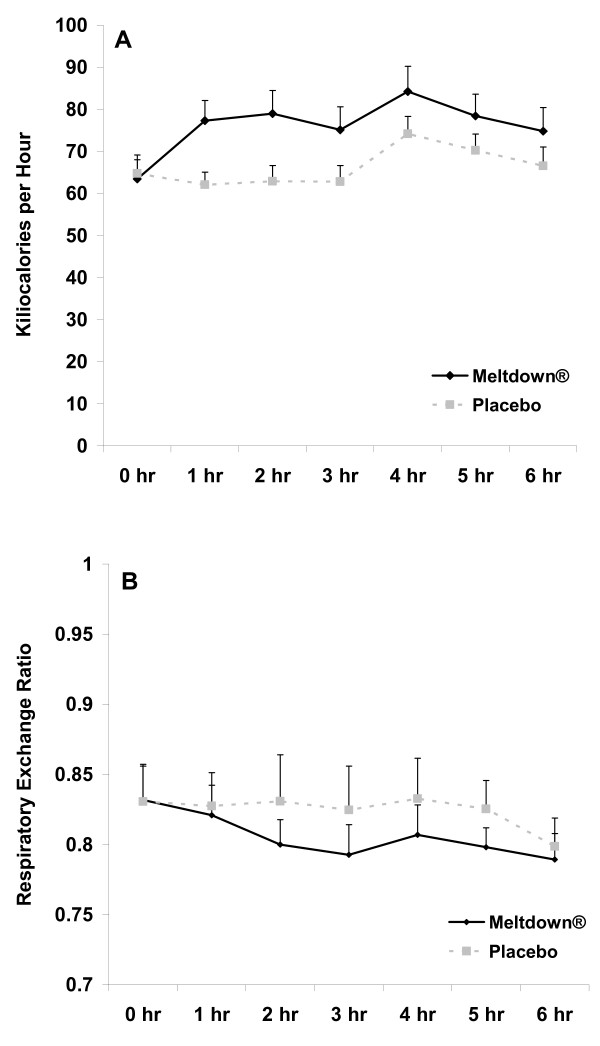
**Kilocalories (A) and respiratory exchange ratio (B) data for men and women consuming Meltdown^® ^and placebo in a randomized cross-over design**. Data are mean ± SEM. * Greater kilocalories (439 ± 26 kcal·6 hrs^-1 ^vs. 380 ± 14 kcal·6 hrs^-1^; p = 0.02) for Meltdown^® ^compared to placebo.

When considering the AUC analysis, no sex × condition interactions were noted for kilocalories (p = 0.27) or RER (p = 0.87). Likewise, when considering the 2 (sex) × 2 (condition) × 7 (time) RMANOVA, no three way interactions were noted (p > 0.05). Therefore, data are presented as pooled data for men and women in Figure 4. Regarding the AUC main effect analyses, both a condition (p = 0.02; Figure 4A) and sex (p < 0.001) effect was noted for kilocalorie expenditure, with values higher for men compared to women. The mean kilocalorie AUC response for Meltdown^® ^compared to placebo was 19% for men and 13% for women. Values increased across time with Meltdown^® ^and were higher at 1, 2, 3, 4, and 5 hours post ingestion compared to pre-ingestion (p < 0.05). Neither a condition (p = 0.39; Figure 4B) nor a sex (p < 0.53) effect was noted for RER.

### Hemodynamic Data

No three way interactions were noted for heart rate, systolic or diastolic blood pressure (p > 0.05). A condition effect was noted for heart rate (p = 0.01), with values higher with Meltdown^® ^compared to placebo. No other effects were noted for heart rate (p > 0.05). A condition × time (p = 0.03) and sex (p < 0.0001) effect was noted for systolic blood pressure, with values higher with Meltdown^® ^across time, and higher for men compared to women. As with systolic blood pressure, a condition × time (p = 0.05) and sex (p < 0.0001) effect was noted for diastolic blood pressure, with values higher with Meltdown^® ^across time, and higher for men compared to women. Hemodynamic data are presented in Table 2.

**Table 2:** Hemodynamic data for men and women consuming Meltdown^® ^and placebo in a randomized cross-over design.

Variable	Sex	Pre	1 hr	2 hr	3 hr	4 hr	5 hr	6 hr
HR (bpm) *Meltdown^®^*	Men	54 ± 3	54 ± 3	62 ± 7	59 ± 4	63 ± 4	62 ± 3	60 ± 2
HR (bpm) *Placebo*	Men	56 ± 4	53 ± 3	53 ± 3	51 ± 3	60 ± 2	57 ± 2	54 ± 3
HR (bpm) *Meltdown^®^*	Women	61 ± 4	56 ± 3	61 ± 4	61 ± 4	61 ± 3	65 ± 6	59 ± 3
HR (bpm) *Placebo*	Women	62 ± 4	57 ± 3	56 ± 4	57 ± 4	59 ± 4	59 ± 4	56 ± 3

SBP (mmHg) *Meltdown^®^*	Men	118 ± 2	128 ± 3	131 ± 5	132 ± 4	131 ± 3	128 ± 3	126 ± 3
SBP (mmHg) *Placebo*	Men	117 ± 3	117 ± 4	115 ± 2	116 ± 3	123 ± 2	117 ± 2	117 ± 3
SBP (mmHg) *Meltdown^®^*	Women	104 ± 3	110 ± 2	114 ± 3	116 ± 3	112 ± 3	109 ± 2	109 ± 2
SBP (mmHg) *Placebo*	Women	104 ± 2	103 ± 2	107 ± 3	105 ± 2	107 ± 2	102 ± 2	104 ± 2

DBP (mmHg) *Meltdown^®^*	Men	74 ± 2	74 ± 2	74 ± 2	78 ± 2	71 ± 3	74 ± 3	72 ± 3
DBP (mmHg) *Placebo*	Men	69 ± 2	71 ± 2	69 ± 2	68 ± 2	67 ± 2	65 ± 2	67 ± 2
DBP (mmHg) *Meltdown^®^*	Women	64 ± 2	68 ± 2	71 ± 2	71 ± 2	66 ± 2	69 ± 1	69 ± 2
DBP (mmHg) *Placebo*	Women	69 ± 2	68 ± 2	68 ± 2	66 ± 2	65 ± 2	61 ± 2	63 ± 2

Data are mean ± SEM.

Condition effect for heart rate (p = 0.01); Condition × time (p = 0.03) and sex (p < 0.0001) effect for systolic blood pressure; Condition × time (p = 0.05) and sex (p < 0.0001) effect for diastolic blood pressure.

HR-heart rate; SBP-systolic blood pressure; DBP-diastolic blood pressure

## Discussion

Our data indicate that the OTC dietary supplement Meltdown^®^, ingested at the exact dosage as recommended by the manufacturer, results in an increase in plasma EPI, NE, glycerol and FFA, as well as an increase in metabolic rate. The increase for all variables is most robust during the initial three hours following ingestion (prior to food intake), but the metabolic rate remains elevated in a statistically significant manner above pre-ingestion values during the five hour post ingestion period. The overall response for our outcome measures is similar for both men and women. An increase in heart rate (ranging from 1–9 bpm), systolic (ranging from 5–16 mmHg), and diastolic (ranging from 1–10 mmHg) blood pressure is also noted with treatment, highlighting the need for careful consideration of use of this agent for those individuals who are hypertensive (blood pressure ≥ 140/90 mmHg) or who may be pre-hypertensive (blood pressure 120–139/80–89 mmHg). For such individuals, it would be best to attempt weight/fat loss through both increased physical activity and modified dietary intake. Once a stable weight is maintained and the individual has a blood pressure value within desired limits, they may consider using such an agent in an attempt for further weight/fat loss. As with any weight/fat loss aid that impacts catecholamine secretion, it is prudent for individuals to be monitored by a qualified health care provider during the course of use.

The current data extend our initial findings in which we noted an increase in plasma EPI, NE, glycerol, FFA, and metabolic rate in a sample of exercise-trained men [8]. In that original investigation of Meltdown^®^, measurements were only carried out for 90 minutes post ingestion, with values for blood borne variables peaking at the 90 minute post ingestion time. The present study provides additional support for the lipolytic effects of this agent, demonstrating that the effects are longer lasting. However, despite relatively high values at the hour three measurement time, values were drastically reduced following intake of food (Figures 2 and 3). This clearly demonstrates the effect of feeding on blunting the catecholamine response, and subsequent lipolysis, associated with lipolytic agents such as Meltdown^®^.

Our findings are similar for both men and women. Hence, both sexes may benefit from use of this agent. While data from our acute studies appear to indicate a potential benefit of this agent on weight/fat loss over time, and anecdotal reports suggest this to be the case, we must admit that no controlled scientific investigations have been conducted testing the chronic effect of this agent on body weight/fat loss, in addition to other important metabolic and biochemical variables associated with obesity (e.g., blood lipids, inflammatory biomarkers, oxidative stress biomarkers, etc.). Future intervention studies are needed to provide evidence pertaining to these outcome measures.

Although we are not certain as to which of the active ingredients contained within Meltdown^® ^are actually responsible for the observed effects, we believe that our findings can be largely attributed to the three primary active ingredients yohimbine, caffeine, and synephrine. Fatty acid oxidation involves the complex interaction between hormone sensitive lipase (HSL), hormones acting to stimulate HSL, and the receptors that bind these hormones in order for them to exert their effect [12]. While hormones such as growth hormone, thyroid hormone, ACTH, and cortisol all appear involved in lipolysis, the catecholamines EPI and NE appear most important [12], as these interact with both beta adrenergic receptors (EPI and NE), as well as alpha-adrenergic receptors (NE). Although HSL is stimulated by the increase in EPI and NE, it is the initial binding of the EPI and NE to beta receptors that begins the secondary intracellular activation of adenylyl cyclase [13]. This results in an increased production of cAMP [14], which leads to the activation of a cAMP dependent protein kinase (PKA) [15]. It is PKA that then activates HSL leading to triglyceride breakdown and subsequent release of glycerol and FFA into the circulation. This was clearly demonstrated in Figure 3. It appears that NE may have the greatest effect on lipolysis [16], which may occur not only via activation of HSL, but through HSL translocation from the cytosol to the lipid droplets in fat cells [17]. Our data support this notion, as the percent increase in NE was more similar to both glycerol and FFA than was the percent increase in EPI (Figures 2 and 3).

Yohimbine itself has been reported in several studies to increase blood NE [18-21], as well as EPI [21,22]. This was clearly demonstrated in the present investigation (Figure 2). This was observed despite the relatively low dosage of yohimbine provided within the supplement (9 mg) compared to other studies using dosages equal to 2–5 times this amount [18-21]. The form of yohimbine used in the supplement could be responsible for the observed increase in EPI and NE, as a combination of yohimbine HCl, alpha-yohimbine, and 11-hydroxy yohimbine make up the total yohimbine complex provided in Meltdown^®^.

Caffeine has lipolytic and thermogenic effects due to its ability to lessen the degradation of cAMP as well as increase cAMP production via beta-adrenergic receptor independent and dependent mechanisms, respectively [23]. The independent effects appear due to caffeine's ability to directly inhibit cAMP degradation, by inhibiting the cyclic nucleotide phosphodiesterase [24] and blocking adenosine receptors. The direct effect results from an increase in catecholamine release following caffeine ingestion, which may be secondary to the previously described adenosine inhibition [23]. Synephrine also interacts with beta receptors (3 sub-class) and promotes lipolysis via the above described cAMP dependent mechanism [25].

Several other ingredients are included within Meltdown^® ^including the amphetamine-like/thyroid stimulating agent phenylethylamine (PEA), which is known to stimulate a rise in blood catecholamine levels and inhibiting their reuptake [26]. The monoamine oxidase inhibitor methyl hordinine is also contained within this supplement. Hordinine is structurally similar to EPI and has been shown to liberate NE from its storage site, in addition to inhibiting NE metabolism [27]. Methyl tetradecylthioacetic acid is also included, and known to stimulate beta oxidation [28] and to be involved in lipid transport and utilization [29].

Collectively, the above ingredients appear to represent a substantial list of potentially effective lipolytic agents. Our data indicate that during an acute monitoring period, Meltdown^® ^effectively stimulates catecholamine secretion, lipolysis, and metabolic rate, and does so in both men and women. Two other studies using Meltdown^®^, while not measuring blood catecholamine levels and markers of lipolysis, support our findings of increased metabolic rate both at rest [30,31] and in response to acute aerobic exercise [31].

## Conclusion

In conclusion, we report that the finished product Meltdown^®^, ingested at the recommended dosage by young and healthy men and women, results in an increase in plasma EPI, NE, glycerol, and FFA, in addition to metabolic rate. This occurs primarily over the initial three hour period following ingestion; however the effects for kilocalorie expenditure are maintained for five hours post ingestion. An increase in both heart rate and blood pressure are also observed during these times, which may be of concern to some individuals, in particular those with elevated resting blood pressure. Whether or not the lipolytic effects are maintained with chronic intake remains to be determined, as most individuals experience some desensitization with chronic treatment, which often requires a higher dosage in order to maintain effectiveness. Moreover, due to the potent metabolic effects of such dietary agents, it is possible that individuals who cease use after chronic intake may experience a lower resting metabolic rate as a result. Future research is needed to address these issues. While anecdotal evidence indicates that Meltdown^® ^continues to exhibit potent effects on weight loss despite intake over periods of several weeks of use, well-controlled clinical trials are indeed needed to confirm this. Such studies may seek to determine the chronic effects of Meltdown^® ^on body weight/fat loss and associated metabolic and biochemical markers of health. Favorable findings could lead to recommendations for the inclusion of this supplement in the weight/fat loss arsenal of those classified as obese.

## Competing interests

Financial support for this work was provided in part by Vital Pharmaceuticals, Inc. Although the authors or the University of Memphis do not directly endorse the dietary supplement, the lead author (RJB) has been involved in scientific writing for Vital Pharmaceuticals, Inc.

## Authors' contributions

RJB was responsible for the study design, biochemical work, statistical analyses, and manuscript preparation; REC, MMB, KGH, and KHFW were responsible for data collection, blood collection and processing; BKS was responsible for the study design and manuscript preparation. All authors read and approved of the final manuscript.
